# Researcher Perspectives on Ethical Considerations in Adaptive Deep Brain Stimulation Trials

**DOI:** 10.3389/fnhum.2020.578695

**Published:** 2020-11-12

**Authors:** Katrina A. Muñoz, Kristin Kostick, Clarissa Sanchez, Lavina Kalwani, Laura Torgerson, Rebecca Hsu, Demetrio Sierra-Mercado, Jill O. Robinson, Simon Outram, Barbara A. Koenig, Stacey Pereira, Amy McGuire, Peter Zuk, Gabriel Lázaro-Muñoz

**Affiliations:** ^1^Center for Medical Ethics and Health Policy, Baylor College of Medicine, Houston, TX, United States; ^2^Department of Neuroscience, Rice University, Houston, TX, United States; ^3^Evans School of Public Policy & Governance, University of Washington, Seattle, WA, United States; ^4^Department of Anatomy & Neurobiology, University of Puerto Rico School of Medicine, San Juan, Puerto Rico; ^5^Program in Bioethics, University of California, San Francisco, San Francisco, CA, United States

**Keywords:** ethics, neuroethics, bioethics, interviews, neuromodulation, deep brain stimulation, ELSI, closed-loop

## Abstract

Interest and investment in closed-loop or adaptive deep brain stimulation (aDBS) systems have quickly expanded due to this neurotechnology’s potential to more safely and effectively treat refractory movement and psychiatric disorders compared to conventional DBS. A large neuroethics literature outlines potential ethical concerns about conventional DBS and aDBS systems. Few studies, however, have examined stakeholder perspectives about ethical issues in aDBS research and other next-generation DBS devices. To help fill this gap, we conducted semi-structured interviews with researchers involved in aDBS trials (*n* = 23) to gain insight into the most pressing ethical questions in aDBS research and any concerns about specific features of aDBS devices, including devices’ ability to measure brain activity, automatically adjust stimulation, and store neural data. Using thematic content analysis, we identified 8 central themes in researcher responses. The need to measure and store neural data for aDBS raised concerns among researchers about *data privacy and security* issues (noted by 91% of researchers), including the avoidance of unintended or unwanted third-party access to data. Researchers reflected on the *risks and safety* (83%) of aDBS due to the experimental nature of automatically modulating then observing stimulation effects outside a controlled clinical setting and in relation to need for surgical battery changes. Researchers also stressed the importance of ensuring *informed consent and adequate patient understanding* (74%). Concerns related to *automaticity and device programming* (65%) were discussed, including current uncertainties about biomarker validity. Additionally, researchers discussed the potential impacts of automatic stimulation on patients’ *autonomy and control over stimulation* (57%). Lastly, researchers discussed concerns related to *patient selection* (defining criteria for candidacy) (39%), challenges of ensuring *post-trial access to care and device maintenance* (39%), and potential effects on *personality and identity* (30%). To help address researcher concerns, we discuss the need to minimize cybersecurity vulnerabilities, advance biomarker validity, promote the balance of device control between patients and clinicians, and enhance ongoing informed consent. The findings from this study will help inform policies that will maximize the benefits and minimize potential harms of aDBS and other next-generation DBS devices.

## Introduction

Adaptive deep brain stimulation (aDBS) devices are part of the emerging field of personalized neurointerventions that are responsive to a patient’s neural activity. In contrast to conventional DBS, the promise of aDBS systems is that they will identify neural activity associated with symptoms and adjust stimulation delivery in real time to alter neural activity and manage symptoms accordingly (Arlotti et al., 2016; Shute et al., 2016). The goal of aDBS systems is to deliver stimulation only when pathological brain activity is detected in order to prevent overtreatment, decrease side effects (e.g., hypomania), and battery depletion, which requires surgical replacement (Hosain et al., 2014; Beudel and Brown, 2016; Shukla et al., 2017). In addition to these safety advantages, aDBS may lead to better outcomes for patients because it adjusts automatically, thus avoiding the delay between suboptimal symptom management and adjustment of stimulation in a clinical encounter (Klein, 2020, p.336).

However, some have suggested that these defining features, which make aDBS promising, may also exacerbate certain neuroethics concerns (Klein, 2020, p.336; Aggarwal and Chugh, 2020, p.158). In particular, aDBS could exacerbate concerns about felt authenticity of affective states and patient agency due to the fact devices adjust stimulation automatically, which likely occur outside of a patient’s conscious awareness (Gilbert et al., 2018, p.9; Gilbert et al., 2018, p.323–324; Goering et al., 2017, p.59–70). Moreover, advancements in aDBS technology depend largely on measuring and storing neural data for programming, raising novel challenges related to patient privacy. Addressing ethical concerns related to these defining features of closed-loop DBS may help to promote safety and efficacy, with potentially broader implications for other next-generation DBS devices containing with similar features.

In an effort to understand researchers’ perspectives on the key neuroethics considerations related to the development of aDBS devices, we conducted interviews with researchers working in aDBS studies, who provided critical insights into the concerns raised by the capabilities and limitations of these devices. Drawing from these interviews, we identify pressing neuroethics issues and concerns, some of which apply to conventional DBS, but many of which are distinctive of or exacerbated by aDBS devices. We contextualize these findings within the existing neuroethics literature and discuss potential responses to these concerns as technologies with adaptive features become more prevalent in the future.

## Materials and Methods

We interviewed researchers (*n* = 23) involved in aDBS trials using a semi-structured, open-ended interview format. Understanding this stakeholder group’s perspectives about ethical issues related to the development of aDBS systems is essential because these individuals possess expert knowledge about these devices, have direct experience developing and implementing them, and/or have expertise related to conditions with characteristics (e.g., treatment-resistance, severity of symptoms) that are similar to the intended users of these technologies. Thus, they are in an ideal position to identify ethical issues and inform resultant discussions related to these devices (Lázaro-Muñoz et al., 2019).

Participants were recruited from funded aDBS trials. Purposeful sampling with a snowball strategy was employed (Palinkas et al., 2015) in order to ensure recruitment of different project roles of researchers involved in aDBS trials (e.g., trial coordinators, neurologists, neurosurgeons, psychiatrics, and engineers) (See Table 1). Our sample also represents a diverse group of researchers targeting different disorders, including Parkinson’s disease, dystonia, essential tremor, Tourette syndrome, depression, and obsessive-compulsive disorder (OCD). One participant was not specifically involved in aDBS but in conventional DBS and other next-generation DBS.

**TABLE 1:** Demographic information of respondents (*n* = 23) involved in aDBS research trials.

Gender (*n* = 23)		
	Male	13 (57%)
	Female	9 (39%)
	Prefer not to answer	1 (4%)
**Race/Ethnicity (*n* = 23)**		
	Asian	3 (13%)
	White	18 (78%)
	Prefer not to answer	2 (9%)
**What degree(s) do you currently hold? (*n* = 23)**		
	M.D. or equivalent	8 (35%)
	Ph.D. or equivalent (clinical)	3 (13%)
	Ph.D. or equivalent (research)	4 (17%)
	Both M.D. and Ph.D. or equivalent (clinical)	2 (9%)
	Both M.D. and Ph.D. or equivalent (research)	1 (4%)
	B.Eng. or M.Sc. Engineering	2 (9%)
	B.A. or B.S.	3 (13%)

Participants were asked about their perspectives on pressing ethical issues in aDBS research and challenges they personally face in their research. We also asked researchers specifically about concerns pertaining to distinctive features of aDBS devices, including the device’s ability to measure brain activity, automatically adjust stimulation, and store neural data. Our interview guide was developed based on a review of key issues raised in bioethics and neuroethics literature, during participant observation in a lab conducting aDBS research, and in discussions with experts in the aDBS field. Respondents were also asked questions about other topics, including several questions related to aDBS data sharing. We report those results elsewhere (Zuk et al., unpublished). The study was approved by the Institutional Review Board at Baylor College of Medicine.

Interviews were conducted via phone and Zoom, and were audio-recorded, transcribed verbatim, and analyzed using MAXQDA 2018 software (Kuckartz, 2014). Each interview transcript was coded independently by at least two members of the research team to identify researcher responses to six questions related to neuroethical concerns in aDBS research. Inconsistencies in coding were discussed to reach consensus among the research team. Utilizing thematic content analysis (Boyatzis, 1998; Schilling, 2006, p.28–37), information from coded segments was progressively abstracted to identify the content and frequency of emergent themes.

## Results

We identified eight overarching themes in researchers’ responses to six questions about neuroethical concerns and challenges in aDBS research (Table 2). Starting with the most frequent, these include concerns related to (1) data privacy and security (noted by 91% of researchers); (2) risks and safety (83%); (3) informed consent and adequate patient understanding (74%); (4) automaticity and device programming (65%); (5) patient autonomy and control over stimulation (57%); (6) patient selection for aDBS candidacy (39%); (7) post-trial access to care and device maintenance (39%); (8) and potential effects on personality and identity (30%). While some of these ethical concerns may be broadly relevant to both conventional and adaptive DBS, most were identified by our respondents as being exacerbated by certain characteristics distinctive of aDBS, particularly its capacity to measure and store brain activity and to respond using automatic stimulation. The ways in which these concerns were specifically raised in response to our six questions is illustrated in Table 2 and elaborated below.

**TABLE 2:** Percentage (%) of respondents (*n* = 23) who discussed main ethical concerns related to aDBS.

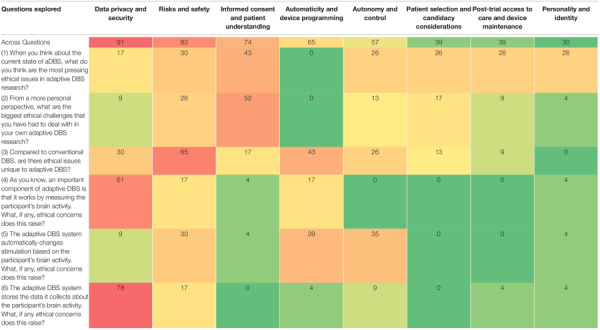

*Thematic percentages do not sum to overall totals due to overlapping concerns raised across multiple questions (i.e., a researcher who raised the same thematic concern in response to multiple questions was only counted once in overall total).*

### Data Privacy and Security

Nearly all (91%) respondents expressed concerns about data privacy and security in relation to the capacity of aDBS systems to measure and store neural activity data (NAD). There was disagreement about the sensitivity of NAD. Some researchers felt that “brain recordings themselves [are] not identifiable” (R_013) because researchers currently do not know enough about what the recordings mean to be able to identify sensitive information, however, this could change in the future (See Table 3). Researchers also pointed out that stored data could be inappropriately used or shared: “The fact that we have the ability to report this data suggests that perhaps it can be used as evidence. Could this be forensic evidence that’s used in lawsuits, in courts, or to settle discussions or arguments?” (R_022). We explored researchers’ views on the sensitivity of NAD as they relate specifically to data sharing elsewhere (see also Zuk et al., unpublished; Naufel and Klein, 2020).

**TABLE 3:** Researcher responses across main ethical concerns related to aDBS.

Ethical theme	Researcher responses
*Data privacy and security*	“I think the main concerns would be privacy of the data. We stream these data to external computers. Someone’s brain data is now […] it could be considered personal health information, in a way. Eventually, we may be able to decode specific things about that person’s identity and personality from their brain data. So, we do have to consider it as personal health information, even if it’s de-identified. At least if not now, then in the future, we’ll have to consider that” (R_011).
*Risks and safety*	“There’s the fact that we just don’t know that much about DBS and how it works. That’s the danger of doing any kind of experiment on humans directly, even though it’s pretty well understood, what the random risks are” (R_006).
*Informed consent and patient understanding*	“In many cases, the person who has a therapeutic relationship with the patient is also an investigator, and so there might be possible duress or coercion to participate in these studies” (R_023).
*Automaticity and device programming*	“My concern is that it might stimulate when it’s not supposed to, causing [an] unwanted side effect. Or the opposite, if it’s not stimulating when it’s supposed to causing the patient unnecessary suffering. Those are glitches that, as we develop these techniques, hopefully will not be an issue. But those are concerns that I have from an ethical perspective. And then, from a researcher point of view and a clinician, when is it going to be that moment [when] we’re satisfied with that signal and that response to stimulation” (R_020).
*Autonomy and control*	“I think we need to be careful in affording control of the device to the patient. For any stimulation of the reward system, there’s potential for self-abuse. There are restrictions [where] patients can turn the device off or on, but they can’t modulate it. That strikes me as a wise precaution” (R_026).
*Patient selection and candidacy considerations*	“When you have a population that does not have a sufficient response to pretty much everything [other treatments], and you can have a 60% response rate in that group [to aDBS], good lord, that’s incredible. I worry about the side effects of *not* doing something for those individuals” (R_018).
*Post-trial access to care and device maintenance*	“We basically thought, ethically, it would be best that they receive rechargeable non-sensing devices so that they can basically get this open-loop therapy for a long duration. I think those batteries last for like 9 years” (R_016).
*Personality and identity*	“In the study where we’re manipulating mood potentially, the goal is to improve mood, which most people would say would be a good thing. But then at some point, do you give somebody a new mood that changes their personality? There are a lot of ethical issues behind potentially manipulating people’s mood and personality [be]cause that could be a good thing or a bad thing” (R_010).

Researchers also discussed device “hacking,” including the potential for stored data or algorithms to be manipulated to disrupt therapy or control patients. One researcher suggested, “We’d have to make sure that there are lots of safety measures in place [.] so that the algorithm can’t be adjusted. Or if we have someone controlling stimulations remotely, like the clinicians. if someone were to steal that control and send the person into a manic state or something maliciously, that would be really bad” (R_017). Some respondents felt that data security risks are minimal because aDBS systems are HIPAA-compliant and researchers who study them are required to submit plans to protect patient information to the FDA. Others, however, emphasized that data security risks could grow as researchers learn more about recordings, and that further plans should be put in place to anticipate future challenges in protecting NAD.

### Risks and Safety

Most (83%) respondents raised ethical challenges surrounding risks and safety, particularly in relation to unique features of aDBS devices (i.e., capacity to measure and store neural activity and automatically adjust stimulation) compared to conventional DBS. In some cases, researchers are inserting additional electrodes in different brain regions (cortical and subcortical) to identify biomarkers that allow for automatic or responsive stimulation. One researcher explained, “Whenever we are pushing the envelope of neuromodulation with new, additional implanted devices, [there is] increased risk of hemorrhage, seizure, stroke, any kind of additional manipulation or extension of the surgery. So, I always have to weigh what the risks and benefits [are] for this specific person” (R_021). A number of researchers discussed unknown risks and unintended effects of aDBS, particularly in relation to automatic delivery of stimulation in new environments outside of the controlled clinical setting (See Table 3). One researcher wondered, “Are there any spot gaps that need to be in place in certain contextual situations that it could fire and do something in a way that we haven’t imagined yet? We haven’t actually thought through and imagined all the potential situations that could play out” (R_015). Unanticipated effects were especially concerning because researchers do not constantly monitor devices, which also raises the “challenge of when to intervene as a clinician taking care of this patient [when] these systems are supposed to be autonomous” (R_020). As a way to potentially mitigate unforeseen risks, respondents emphasized the importance of working within safe stimulation parameters and maintaining researchers’ ability to intervene when necessary.

Other researchers raised concerns that stimulation could inadvertently and unknowingly affect other neural circuits, potentially causing side effects. The risk of overlooking these side effects may be exacerbated in aDBS because researchers – and aDBS systems operating autonomously – could be overly focused on therapeutic outcomes. As one researcher described, “We’ve always looked at therapeutic outcomes, but then became increasingly aware of the side effects… So the potential is to cause more side effects unknowingly, especially in an adaptive system that’s not tuned to the right outcome” (R_023). Respondents also discussed risks specific to certain subpopulations of aDBS patients, such as overstimulation leading to hypomanic states in patients with OCD.

### Informed Consent and Patient Understanding

A majority (74%) of researchers said that one of the most pressing ethical concerns in aDBS studies is ensuring that patients understand and are able to provide informed consent to aDBS. Over half of respondents reported having encountered related challenges in their own aDBS research. Some researchers raised the concern that patients may feel pressured to participate, particularly because “some of the patients who are looking at this kind of procedure don’t really have other helpful interventions” (R_005). Patients may also feel pressured to participate in aDBS research due to an established therapeutic relationship with clinician-researchers leading aDBS research studies (See Table 3). Further potential compromises to informed consent can stem from therapeutic misconception and therapeutic misestimation. One respondent explained patients can potentially “lose track of the investigational nature of the study” (R_011), and another respondent suggested that it is challenging to ensure realistic patient expectations about aDBS during the consent process: “DBS…seems, to them – because it’s so risky, but can have such promise – that it’s like a silver bullet, so to speak” (R_005). Patients must understand that aDBS is a complex intervention and not “one size fits all” (R_015).

Researchers also reflected on how the automatic nature of aDBS raises unique ethical concerns about patient consent. One researcher wondered whether patients can robustly consent to automatic, moment-to-moment changes in stimulation, explaining that “it’s almost as though the intervention is changing at each time point” (R_018). Researchers stressed the need to ensure that patients who explicitly consent to the adaptive component of aDBS at the beginning of their treatment are continuing to implicitly consent to ongoing stimulation changes, which evolve as device recognition of a patient’s neural activity improves and likely occur outside of a patient’s conscious awareness. To address this concern about patient consent, one researcher suggested that devices could be designed to notify patients when they detect symptom-related brain activity: “It would be interesting to have a device be able to [.] give the patient an alert somehow. ‘[If] I [the device] think you’re dyskinetic, I’m going to turn myself down.’ The patient could override it” (R_013). Researchers conveyed that improving patient understanding about when and how the device adjusts stimulation can help to ensure that patients are continuing to consent to the device’s automatic changes. Consent challenges may be especially pronounced among certain subpopulations of patients, including those with severe psychiatric symptoms that potentially influence decisional capacity.

### Automaticity and Device Programming

Researchers (65%) raised unique concerns related to automaticity and device programming for aDBS systems. They stressed the importance of using validated, reliable biomarkers given that researchers are relying upon a device designed to make autonomous decisions to affect patients’ mood, behavior, or movement. One researcher wondered, “How validated does a biomarker have to be before you start deploying a system like this and letting it deliver therapy in real time?” (R_023). Researchers explained that a biomarker lacking validity could cause devices to respond to false positives or negatives, leading to over- or under-stimulation. These “misreadings” of neural biomarkers could result in patients experiencing suboptimal symptom management or undesirable effects (See Table 3). One researcher shared,

“Let’s say we come across… a good biomarker for hypomania, and it misreads the patient just having a really great weekend, because they’re at a family member’s wedding. Now all of a sudden, they’re depressed again or they’re feeling more of their OCD symptoms come on at that time. That’s obviously a problematic situation we want to avoid” (R_007).

Respondents said that to avoid stimulation errors, devices would ideally be programmed so they could recognize when “the patient’s behavior and mood is elevated beyond where it is beneficial to the patient” and subsequently “turn down the system” (R_019). Researchers were also concerned that patients may be unaware of inappropriate stimulation changes because the changes are occurring automatically, impacting patients’ and clinicians’ ability to actively intervene to mitigate negative consequences. As one researcher described, “There is still a decision being made on a second-by-second basis out in the field, in the wild, by an algorithm that may change that person’s current mental status” (R_025). Another researcher stressed the ethical implications of this unique feature of aDBS, saying, “[Normally], we always have a physician intervening and assessing, [but aDBS] is an autonomous system making decisions about the delivery of therapy” (R_023).

### Autonomy and Control

Related to the concerns about automaticity described above, over half (57%) of respondents raised concerns related to patient autonomy and control over stimulation. One researcher explained how “people’s sense of autonomy may be altered by the use of a computer unit” if they believe their “motor state or their mental state… are being controlled by an external source or by a computer” (R_008). Researchers felt this concern could be particularly exacerbated for aDBS patients due to the fact that changes in stimulation occur automatically. Another researcher commented, imagining from a patient’s perspective, “Even with open loop, there’s the issue that now I have a device in my brain that’s modulating and controlling some of my brain activity. I think as we develop closed-loop, that concern about allowing a device to take some command over your activity will be extenuated” (R_009). This researcher speculated that automatic device control may be even more concerning for psychiatric patients if they view the targets of aDBS adjustment – e.g., mood and anxiety – as central to their sense of self and identity. They said, imagining the perspective of a patient, “’A tremor doesn’t represent me. It’s a dysfunction.’ [But] when you have a device that’s modulating your mood or your anxiety level, your energy level, that’s much more your core sense of being” (R_009) (See also Personality and Identity below).

Alternatively, one researcher noted, “[patients] seem to have an awareness of how the device is being set. They trust the researchers that are controlling it. They don’t feel like there’s any questionable agency to be concerned with” (R_008). According to another researcher, concerns about patients’ sense of control, “are mitigated substantially by the design of these protocols, where patients do have a controller and at any point can flip themselves out of adaptive stimulation into conventional stimulation” (R_011). While some researchers highlighted this need to allow patients to override unwanted stimulation, others alluded to potential risks of giving patients substantive control over their stimulation. Some respondents noted that determining how much control patients should have over stimulation may depend on which areas of the brain are being stimulated. In cases like aDBS for OCD, in which part of the brain’s reward circuit is stimulated, some researchers said they feel hesitant providing too much patient control due to the potential for stimulation abuse (See Table 3). Researchers said that other patients, such as those with essential tremor who receive stimulation elsewhere in the brain, could be given greater unilateral discretion to adjust stimulation. Overall, researchers stressed caution in deciding whether and how much patient control to allow.

Some researchers offered similar cautions against giving physicians too much control, advocating for limits to physician access to stimulation. As one respondent commented, “We still don’t want the clinician to be kind of messing with it whenever they want to. How do you put in the safeguard so that only authorized people can access it, and even they can only do so with the patient’s permission every time?” (R_022). Another respondent highlighted a tension between ensuring patient safety and respecting their autonomy, saying, “In the future, it would be important to have a button that the doctor could press remotely if they hear something is going on, like turn everything off or turn it down…But then that’s like a doctor controlling remotely” (R_017). One researcher suggested that a potential solution to finding an ethical balance is to integrate all stakeholder groups – including patients and caregivers – in the development of control and safety policies.

### Patient Selection and Candidacy Considerations

Over a third of researchers (39%) raised ethical concerns related to patient selection and candidacy for aDBS treatment. Because DBS treatment is an invasive therapy typically offered to patients who are treatment-resistant, some researchers said they want to be sure that patients have “tried enough different treatments, even some of the ones that are a bit more experimental” (R_009) in order to warrant taking on the challenges and risks of aDBS. Other researchers noted that the treatment-resistant nature of a patient’s disorder supports not only their fit as an aDBS candidate but also the ethical imperative to make aDBS treatment available to them (See Table 3). Respondents pointed out that deciding whether and when a patient may benefit from aDBS requires that multiple clinical and demographic factors be taken into consideration. For example, researchers discussed the difficulty of defining “normal” versus “abnormal” thoughts, moods, and behaviors in the context of aDBS. Ideally, aDBS treatment will be able to appropriately decipher between “normal” and “abnormal,” however, some researchers said it may be problematic to expect a human, let alone a machine to make these fine distinctions. One researcher said,

“I think the most interesting, challenging question to me is, what are we defining as our set point or as our ‘normal?’ I think for a movement disorder [like] tremors, for example, ‘normal’ is not having tremors. When you’re talking about mood and anxiety, like with OCD, how much time you spend thinking about whatever is your concern – contamination or orderliness or symmetry… Is that normal? Is that abnormal?” (R_022).

### Post-Trial Access to Care and Device Maintenance

Nearly a third (30%) considered post-trial access to care and device maintenance to be an additional pressing ethical issue. Researchers said that some patients who want to continue aDBS are unable to access aDBS care and device maintenance after a research study ends: “I think, honestly, the biggest issue right now is the amount of money that it costs patients to maintain the device, or obtain a replacement after the study is over” (R_004). Reasons for this include the fact that conventional DBS and aDBS have not yet been approved by the FDA for some of the conditions targeted in trials, which may result in insurance providers not covering costs associated with battery or hardware replacements, thereby limiting post-trial access. Post-trial access to care may be particularly problematic for certain patients, such as those whose batteries need replacing early. For example, one researcher said, “For Tourette’s therapy, amplitudes are really high, and [batteries] get depleted really quickly. And then it’s not FDA approved, so they don’t get it covered by insurance companies. We can’t promise to provide them again with the study devices, even if they convert to standard DBS batteries” (R_016). Some researchers recommended giving patients conventional DBS with rechargeable batteries at the end of studies to extend these patients’ access to DBS (See Table 3).

### Personality and Identity

Researchers (30%) also discussed the important ethical challenge of mitigating potential unwanted effects of aDBS on personality and identity, including mania or hypomania caused by aDBS stimulation in patients with OCD. One researcher commented, “One [concern] is, changing someone’s personality, and their behavior and how that can be manipulated through deep brain stimulation, either inadvertently or maliciously. That’s one of my concerns” (R_021). Another researcher felt that the brain was a unique organ and different from, for example, the heart. They explained that altering brain activity and “directly stimulating reward tracks in the brain, generat[e] both hedonic responses” and other responses “that are really part of the fabric of personality.” On the other hand, “for someone with heart irregularities, a cardiac pacemaker may be beneficial, expanding their range of motion and activity, but only very indirectly, if at all, affecting them as an individual” (R_026). While manipulating and improving mood could be the goal for some uses of aDBS, researchers expressed concern over lasting changes on personality (See Table 3). Furthermore, navigating these situations could be particularly challenging for researchers in cases where patients do not understand or acknowledge that their mental state is negatively affecting their functioning.

## Discussion

### Minimizing Vulnerabilities in Cybersecurity

In this paper, we identified potential ethical issues and challenges that are heightened in or unique to aDBS research relative to conventional DBS, drawn from the perspectives of aDBS researchers working at the forefront of their field. Our findings suggest that the technical features that give aDBS distinct advantages over conventional DBS systems also raise distinct issues that should be addressed in order to ensure that patients receive the full benefits of these neurotechnologies while minimizing potential medical and non-medical harms. Among the most pressing concerns raised by researchers was the potential for aDBS systems to compromise patient privacy and data security. Researchers pointed out that while NAD that is recorded and stored by aDBS systems may not itself contain identifiers or other sensitive information presently, this could change in the future, which is a concern frequently raised in the theoretical neuroethics literature (Klein, 2016, p.1310; Zuk et al., 2018, p.45–46; Aggarwal and Chugh, 2020, p.160). Theoretical work further predicts that privacy concerns will increase as larger amounts of data are collected, advances in technologies make it easier to integrate data, and DBS devices interface with other devices in the future (Hendriks et al., 2019, p.1508; Klein, 2020, p.335). Researchers should therefore maintain awareness of advances in neuroscience and technology that could change the degree of NAD sensitivity and implement additional data protections if and when necessary. Researchers also have a responsibility to inform participants of what information could and could not be extrapolated from their neural recordings (Pugh et al., 2018, p.221). Moreover, researchers and clinicians will need to determine participants’ desired boundaries around neural privacy and preferences around how their NAD is used in the future, which could require researchers to not collect or to filter certain kinds of neural recordings (Klein, 2020, p.335).

To avoid the possibility of device hacking, data manipulation, and therapy interruption, researchers and clinicians can incorporate additional security patches and upgrade software systems to reinforce the cybersecurity of both hospital-wide networks as well as patient devices linked to networks (Jaret, 2018; Pugh et al., 2018, p.221). The FDA should also hold device manufacturers accountable for identifying and addressing vulnerabilities in medical devices and ensure that the responsibility to safeguard devices is shared amongst providers and manufacturers. Currently, the FDA is exploring the development of a CyberMed Safety (Expert) Analysis Board, which is “a public-private partnership that would complement existing device vulnerability coordination and response mechanisms and serve as a resource for device makers and FDA” (U.S. Food & Drug Administration, 2018). This board would function to assess vulnerabilities, patient safety concerns, and mitigation plans, which could play a large role in supporting aDBS researchers and addressing device security concerns.

### Mitigating Risks and Advancing Biomarker Validity

A second highly salient concern raised by researchers is the need to mitigate risks and ensure safety for patients being treated with aDBS. Identifying valid neurophysiological biomarkers is an enduring challenge for researchers that involves a variety of strategies, depending on the disorder. For example, with essential tremor, researchers record NAD while patients perform a motor task (e.g., clasping a cup and brining it toward their mouth) (Opri et al., 2019). With Parkinson’s Disease, researchers record NAD when patients are on and off medication and on and off therapeutic DBS (Swann et al., 2016).

Identifying biomarkers for psychiatric disorders, however, is especially challenging because there are often no external, visible symptoms as in motor disorders, and psychiatric symptoms involve highly complex and dynamic cognitive states and behaviors. Currently, researchers developing aDBS for OCD can utilize video recording of facial expressions and physiological measurements (e.g., heart rate) collected while patients perform psychophysical tasks (e.g., unscripted social interactions with strangers) (Girard et al., 2015; Provenza et al., 2019). However, the validity of biomarkers identified during these tasks depends on the extent to which they elicit the same brain processes associated with OCD symptomology as it manifests in everyday life. To help improve biomarker validity, particularly for psychiatric disorders, further research is needed into the translatability of clinic-derived biomarkers to neural processing and patient functioning in less controlled and more naturalistic settings (Provenza et al., 2019). This research could help address researchers’ concerns and fill contextual blind spots that could cause aDBS devices to “misread” brain activity and either over- or under-stimulate. Improving biomarkers may also help to mitigate potential unwanted effects of aDBS on personality and identity, another significant concern raised by respondents, by avoiding device settings associated with any such effects.

### Promoting Autonomy and Balancing Device Control

Researchers from our sample recognized that determining when clinicians should intervene to ensure patient safety is challenging for a number of reasons, including that researchers and patients may not be aware of when the device begins stimulating inappropriately, and researchers may feel uneasy about potentially violating patient privacy or undermining patient autonomy. Researchers’ reflections on autonomy and patient control illuminate the challenging and complex nature of these issues and suggest possible tension between patient safety and patient autonomy. On one hand, some researchers suggested that patient autonomy requires that *clinicians* do not have too much control and that *patients* have adequate control over aDBS functionality, such as having the ability to reject an upcoming change in stimulation (Fins, 2009; Goering et al., 2017, p.65). One way to manage this and respect patient autonomy would be to engage patients and clinicians early in the consent process to discuss preferences and conditions for patient versus physician intervention within the larger context of a patient’s treatment goals. Patients could also identify a close caregiver to provide assistance in adjusting stimulation parameters or finding appropriate medical care when clinicians or caregivers identify a concern during treatment, thus supporting patient autonomy in a *relational* way (Baylis, 2013, p.516–519; De Haan et al., 2015, p.22; Goddard, 2017, p.332–334; Goering et al., 2017, p.67; Gallagher, 2018).

On the other hand, some researchers suggested patients may trust or prefer clinicians to have a substantial amount of control, and for certain patients, providing them with too much control could lead to autonomy being undermined. Despite concerns about autonomy and control being raised frequently in theoretical neuroethics and sometimes in empirical work, some researchers believed that, at least in general, autonomy concerns are not highly problematic in the context of aDBS research context because patients trust clinicians to manage treatment modifications (Lipsman and Glannon, 2013, p.468; De Haan et al., 2015, p.6–16; Klein, 2016, p.1311; Gilbert et al., 2017, p.96; Gilbert et al., 2018). A study by Klein in 2016 found that the majority of patients receiving open-loop DBS expressed a preference for primarily clinician-controlled rather than patient-controlled stimulation settings, were such control to become available (Klein, 2016, p.3). Additionally, empirical work indicates that the brain region targeted is also an important consideration when examining potential effects that DBS could have on patient autonomy and control (Gilbert et al., 2017, p.101). Researchers in our sample similarly stated that it would be wise to limit the degree of control of patients with OCD given that they receive stimulation in the reward system (i.e., ventral striatum), which could lead to stimulation abuse. Over-stimulation of this brain region could result in mania and increased risk-taking behaviors, which could alter judgment or diminish the degree of control patients have over their actions, thus undermining autonomy (De Haan et al., 2017, p.23; Gilbert et al., 2017, p.98–99).

One can foresee a potential conflict between the above considerations if, for example, a patient receiving aDBS for OCD in the ventral striatum is limited in their ability to adjust stimulation and feels on that basis that they lack adequate control. These considerations are further complicated by the positive impact of symptom relief, which could outweigh potential diminishments in autonomy resulting from a lack of control over device functionality (Lázaro-Muñoz et al., 2017, p.74). Ideally, a balance between patient and clinician control over stimulation will be achieved through the assessment of individual patient preferences, targeted brain region, and different means of device control. All relevant stakeholders will need to be involved in these discussions, including patients, caregivers, clinicians, programmers, and engineers. This process may be assisted by development of multi-faceted empirical measures incorporating different conceptions of autonomy, which will be particularly useful given patients have been found to use the idea of “becoming a new person” inconsistently and not all researchers in our sample made a clear distinction between autonomy and sense of autonomy (Roskies, 2015, p.6; Sullivan, 2015, p25; De Haan et al., 2017, p.17–18; Zuk and Lázaro-Muñoz, 2019).

### Enhancing Patient Knowledge and Ongoing Informed Consent

Researchers pointed out that issues related to safety and autonomy highlight the need for patients to adequately understand and provide informed consent to aDBS treatment, which is a concern that is frequently raised in empirical and theoretical neuroethics literature (Cabrera et al., 2014, p.37–42; De Haan et al., 2015, p.25; Chiong et al., 2018, p.32–33; Klein, 2020, p.330). Pre- and post-operative counseling and psychosocial support could provide opportunities for patients to learn about aDBS, including how aDBS works, the role and rationale behind automaticity, and what the unique features of aDBS imply for ongoing consent. These forums would provide patients with multiple opportunities to voice any concerns or uncertainties about their treatment so that problems may be mitigated or avoided early on and at different time points throughout a patient’s treatment trajectory (De Haan et al., 2015, p.20).

Severe, refractory symptoms combined with a lack of treatment alternative suggests that patients considering aDBS are in a more vulnerable position than most and may perceive research participation as their only option, a situation that could be further influenced by the presence of a therapeutic relationship between the patient and a study investigator (Cabrera et al., 2014, p.39–42; Chiong et al., 2018, p.32, p.34; Zuk et al., 2018, p.48; Morain et al., 2019, p.11; Klein, 2020, p.333). Researchers shared these same concerns around patient consent and acknowledged that they have a responsibility to ensure that consent is not inadvertently biased by a patients’ perceptions or expectations. More specifically, researchers felt that patients should be adequately informed of aDBS devices’ unique ability to automatically adjust stimulation, which could potentially prevent some autonomy related concerns (Aggarwal and Chugh, 2020, p.156). More research is needed to clarify patient understandings about what they believe they are consenting to when they agree to participate in an aDBS trial, how consent may change over the span of the trial, and how understandings affecting consent may differ among certain patient subpopulations (Chiong et al., 2018, p.33–34).

Adequate patient understanding of aDBS research participation will also require that patients are informed about potential post-study uncertainties and issues (Lázaro-Muñoz et al., 2018, p.317–318; Hendriks et al., 2019, p.1511; Sierra-Mercado et al., 2019, p.760). Researchers expressed the need to help ensure post-trial access to care and device maintenance. They were concerned that patients who wanted to continue DBS may not be able to due to a lack of FDA approval for certain indications, causing insurance providers to not cover costs associated with battery or hardware replacements and clinical visits in some cases. Ensuring that patients understand these potential limitations to post-trial access to aDBS or conventional DBS was viewed by respondents as a critical aspect of informed consent procedures for these trials (Klein, 2016, p.1308). In addition to informing patients of the current realities of post-trial access, ongoing discussions are needed to determine different stakeholders’ obligations and potential responses, such as funders making supplementary funds available and device manufacturers covering costs to help improve post-trial access to care and device maintenance (Zuk et al., 2018, p.46; Hendriks et al., 2019, p.1511).

Our results should be considered within the limitations of our study. The lack of representation of all clinical applications of developing aDBS systems limits the generalizability of our findings. Our sample includes researchers working on aDBS systems for six different disorders, however, a more robust sample size could enhance insights into different uses of aDBS systems and closed-loop devices more generally. Furthermore, the researchers interviewed are experts working on the development of these technologies in a translational research context, thus, their perspectives may not capture the range of ethical considerations that could arise if aDBS systems are adopted more widely in clinical care. Researchers are just one of the key stakeholder groups involved in the development of aDBS systems. Other groups such as patients and caregivers may have different perspectives which are critical to understand to promote the responsible use and development of these technologies. Although we ensured recruitment of researchers who have various professional roles in aDBS trials, 78% of the sample identified as white, reflecting a lack of racial and ethnic representation in our sample, which could be addressed through more purposeful sampling. Other limitations of qualitative research include potential ambiguity in interview responses, which could lead to misinterpretation of data. Thematic content analysis was performed by at least two independent team members and inconsistencies in abstracted coded segments were discussed to reach a consensus among the research team to mitigate the potential impact of this limitation.

## Conclusion

Drawing on the perspectives of expert stakeholders working at the forefront of aDBS research, we identified potential ethical issues and challenges that are heightened in or unique to aDBS research relative to conventional DBS. Due to the need to measure and store neural data, aDBS researchers raised concerns about protecting the privacy of neural data and preventing unwanted third-party access to data. The automatic nature of stimulation sparked risk and safety concerns associated with the experimental nature of identifying biomarkers to automatically adjust stimulation outside the clinic. Additionally, researchers discussed challenges of determining the degree of control researchers and patients should have over adaptive stimulation and challenges of ensuring that patients provide appropriate consent to continuous alterations in stimulation. Our findings therefore suggest that the technical features that give aDBS advantages over conventional DBS systems also raise distinct issues. We identified four areas where researcher concerns can begin to be addressed, including minimizing cybersecurity vulnerabilities, advancing biomarker validity, promoting the balance of device control between patients and clinicians, and enhancing ongoing informed consent. Further research and ethical analysis of these pressing issues are needed to better ensure that patients receive the full benefits of these neurotechnologies while minimizing potential medical and non-medical harms.

## Data Availability Statement

The datasets presented in this article are not readily available because full datasets must remain unavailable in order to ensure de-identification of interview participants.

## Ethics Statement

The studies involving human participants were reviewed and approved by Baylor College of Medicine Institutional Review Board. Written informed consent for participation was not required for this study in accordance with the national legislation and the institutional requirements. Written informed consent was not obtained from the individual(s) for the publication of any potentially identifiable images or data included in this article. Verbal consent was obtained from each research participant before beginning interviews.

## Author Contributions

All authors contributed to project design. SO, LT, DSM, and RH conducted interviews and contributed to data collection. KAM, KK, CS, and LK completed data analysis. RH provided assistance in identifying global themes that emerged during data analysis. KAM, KK, PZ, and GLM conceptualized the manuscript. KAM drafted the manuscript. KK, PZ, and GLM made substantial revisions to the manuscript. SP, JOR, and AM also provided feedback and suggested edits on the manuscript. DSM provided insight on the technical points raised in the paper. SP, AM, GLM, JOR, and BAK provided senior-level leadership for the project.

## Conflict of Interest

The authors declare that the research was conducted in the absence of any commercial or financial relationships that could be construed as a potential conflict of interest.
